# NIH Toolbox Cognition Battery: Associations with plasma and imaging AD biomarkers in older adults without dementia

**DOI:** 10.1002/alz70856_103538

**Published:** 2025-12-26

**Authors:** Jeremy M. Gu, Laisze Lee, Dana L Tudorascu, Rebecca E. Roush, Xuemei Zeng, Ann D Cohen, Thomas K Karikari, Beth E. Snitz

**Affiliations:** ^1^ University of Pittsburgh School of Medicine, Pittsburgh, PA, USA; ^2^ University of Pittsburgh, Pittsburgh, PA, USA

## Abstract

**Background:**

The NIH Toolbox Cognition Battery (NIHTB‐CB) evaluates fluid and crystallized cognitive abilities and has gained traction in AD research. Its relationship with AD biomarkers has been little investigated. This study examined these relationships using novel plasma and imaging biomarkers in older adults without dementia to assess the NIHTB‐CB's sensitivity to early‐stage pathology.

**Methods:**

This study included 258 participants (mean age 63.7 ± 8.9; 66.7% female; 49.2% non‐Hispanic White) from the *Connectomics of Brain Aging* study. Participants underwent ADRC clinical classification, [11C] PiB‐PET imaging, and MRI for cortical thickness (CT). Plasma BD‐tau, *p*‐tau217, *p*‐tau181, *p*‐tau231, Aβ42, Aβ40, GFAP, and NfL were measured using Single Molecule Array assays. Linear regression models assessed relationships between NIHTB‐CB uncorrected standard scores and AD biomarkers, controlling for age, sex, race, and education. Analyses were repeated in subsets: (1) cognitively normal (CN, *n* = 127) and (2) CN+ (CN + impaired with complaints + subjective cognitive decline, *n* = 192).

**Results:**

P‐tau217 (Picture Vocab, List Sorting, Crystal Cognitive Composite, Total Cognitive Composite, p value [0.006, 0.049]; whole sample) and CT (all metrics except List Sorting and Pattern Completion, p value [0.0003, 0.023]; whole sample) showed strongest associations with NIHTB scores over other biomarkers. CT composite had strongest associations in the whole and subset analysis, particularly Fluid (β = 0.254, *p* < 0.001; whole sample) and Total Cognition Composites (β = 0.296, *p* < 0.001; whole sample). CN subset showed lower CT (β = 0.239, *p* =  0.029) and higher PiB‐PET SUVR (β= ‐0.195, *p* =  0.049) and *p*‐tau217 (β = ‐0.242, *p* =  0.017) associated with worse fluid cognitive scores, whereas higher *p*‐tau 181 (β = 0.245, *p* =  0.029) and *p*‐tau 231 (β = 0.268, *p* =  0.013) were associated with better crystallized cognition.

**Conclusion:**

NIHTB‐CB reveals cognition‐biomarker relationships, including plasma biomarkers in cognitively normal individuals. CT is a key marker of neurodegeneration's proximal impact on cognition. Positive associations in the CN subset may reflect selection bias towards cognitive reserve and compensatory mechanisms in early preclinical stages. Findings support NIHTB‐CB's potential for detecting early cognitive changes in AD. Future research will assess its predictive utility for cognitive decline and disease progression.

